# A Combined Frame Difference and Convolution Method for Moving Vehicle Detection in Satellite Videos

**DOI:** 10.3390/s25020306

**Published:** 2025-01-07

**Authors:** Xin Luo, Jiatian Li, Xiaohui A, Yuxi Deng

**Affiliations:** Faculty of Land and Resources Engineering, Kunming University of Science and Technology, Kunming 650093, China; 20222101042@stu.kust.edu.cn (X.L.);

**Keywords:** motion target detection, frame difference method, satellite video, neural network, self-reconfiguration

## Abstract

To address the challenges of missed detections caused by insufficient shape and texture features and blurred boundaries in existing detection methods, this paper introduces a novel moving vehicle detection approach for satellite videos. The proposed method leverages frame difference and convolution to effectively integrate spatiotemporal information. First, a frame difference module (FDM) is designed, combining frame difference and convolution. This module extracts motion features between adjacent frames using frame difference, refines them through backpropagation in the neural network, and integrates them with the current frame to compensate for the missing motion features in single-frame images. Next, the initial features are processed by a backbone network to further extract spatiotemporal feature information. The neck incorporates deformable convolution, which adaptively adjusts convolution kernel sampling positions, optimizing feature representation and enabling effective multiscale information integration. Additionally, shallow large-scale feature maps, which use smaller receptive fields to focus on small targets and reduce background interference, are fed into the detection head. To enhance small-target feature representation, a small-target self-reconstruction module (SR-TOD) is introduced between the neck and the detection head. Experiments using the Jilin-1 satellite video dataset demonstrate that the proposed method outperforms comparison models, significantly reducing missed detections caused by weak color and texture features and blurred boundaries. For the satellite-video moving vehicle detection task, this method achieves notable improvements, with an average F1-score increase of 3.9% and a per-frame processing speed enhancement of 7 s compared to the next best model, DSFNet.

## 1. Introduction

With the rapid advancement in satellite remote sensing technology, motion object detection (MOD) in satellite videos has become a pivotal task for automated satellite monitoring. MOD is to locate and identify objects in video sequences that exhibit semantic similarity and continuous motion across spatial and temporal dimensions, which plays a vital role in object tracking tasks [1]. However, the unique characteristics of satellite video data, including wide fields of view, complex backgrounds, and numerous objects, present significant challenges. Moving vehicles are typically small, exhibit weak texture features, and have low contrast with their background. Target-tracking algorithms designed for ordinary video scenes do not perform as well in satellite video scenes. Therefore, it is necessary to redesign the applicable algorithms for the characteristics of satellite video [2,3,4,5].

Traditional methods for moving object detection (MOD), such as optical flow, background subtraction, and frame difference, are straightforward and effective under controlled conditions. However, they have limitations, including high computational costs, sensitivity to environmental noise and changes in illumination, and poor performance in detecting slow-moving or small targets. Recent advancements in deep learning have led to the development of more sophisticated algorithms that leverage spatiotemporal features and neural networks to enhance detection accuracy. Despite these improvements, these methods often encounter challenges, such as information mixing across frames, difficulties in capturing weak targets, and increased computational complexity, which limits their real-time applicability in satellite video scenarios.

To address these issues, this paper introduces an efficient algorithm for moving target detection in satellite videos. By integrating the frame difference method with convolution, the algorithm optimizes multiframe information processing, thereby enhancing the model’s capability to detect weak targets. This approach effectively reduces missed detections caused by weak color and texture features or blurred boundaries, while maintaining balance between the high performance and real-time requirements of deep learning methods. The major contributions of this paper are as follows.

1. We design a frame difference module (FDM) to effectively extract spatiotemporal features by combining the frame difference method with convolution. The FDM leverages sequential historical frames to extract temporal information, which is subsequently fused with the current frame to generate the initial spatiotemporal features of the current image.
2. We introduce a self-reconstructed tiny object detection (SR-TOD) framework. This framework incorporates a reconstruction head between the detection head and neck, creating a difference map between the reconstructed image and the input image to enhance the weak features of objects under the guidance of the difference maps.
3. For the first time, we introduce an efficient multi-scale attention (EMA) module in the field to optimize the representation of features across consecutive frames. This approach mitigates interframe information blending and enhances detection ability.
4. Experimental results demonstrate that the proposed method effectively detects more weak targets with indistinct color and texture features or blurred boundaries, offering an efficient solution for motion object detection in satellite videos.

The structure of the article is as follows. Section 2 reviews related works. Section 3 provides a detailed description of the proposed method for detecting moving vehicles in satellite videos. Section 4 presents the dataset, experimental setup, and evaluation metrics, followed by a comprehensive analysis of experimental results. Finally, Section 5 concludes the article with a summary and suggestions for future research.

## 2. Related Works

### 2.1. Traditional Object Detection Methods

Traditional approaches for detecting motion in satellite videos typically rely on the physical properties of objects. These methods include optical flow-based, frame difference-based, and background subtraction-based.

Optical flow-based methods [6,7,8] detect moving objects by calculating motion fields for individual pixels across video or image sequences and analyzing temporal variations and correlations within these fields. However, these methods can be computationally expensive, sensitive to lighting changes, and unsuitable for scenarios with significant illumination variations.

Background subtraction-based methods [9] estimate the background using various filters and then subtract it from each frame to obtain detection results. Simpler background estimation techniques often reduce performance in complex scenes, leading to higher false-positive rates. Other background subtraction approaches leverage robust principal component analysis (RPCA) to detect moving objects by decomposing satellite video frames into low-rank background matrices and sparse foreground matrices. These RPCA-based methods [10,11,12,13,14,15] assume that satellite video images consist of background, objects, and noise, applying different regularization terms for each component and using closed-form solutions with iterative optimization for detection. However, these methods often require laborious manual regularization for complex scenes, involve high computational complexity, and process frames slowly. Additionally, maintaining the quality of background reconstruction can be challenging, which compromises detection performance.

Frame difference methods [16,17,18,19,20] detect moving objects by calculating the differences between adjacent frames and segmenting the results. While simple and fast, this approach struggles to detect slow-moving objects and is susceptible to environmental noise. Frame difference is often combined with other methods, such as optical flow, Gaussian mixture models, or background modeling, to reduce false positives caused by lighting changes and background motion.

### 2.2. Object Detection Methods Based on Deep Learning

Deep learning has achieved remarkable performance in object detection for natural images, and researchers have adapted these algorithms for moving object detection in satellite videos. LaLonde et al. [21] proposed a two-stage spatiotemporal convolutional neural network (CNN) that combines motion and appearance information to extract candidate motion regions from ultra-large video frames, followed by heatmap estimation to identify moving objects. Chen et al. [22] introduced a lightweight KNN + LeNet5 framework, where a k-nearest neighbors background subtraction model generates initial detection results, and a shallow CNN suppresses false positives. While simple, this approach heavily relies on the accuracy of background subtraction and exhibits poor real-time performance.

Multiframe information processing has also attracted significant attention. Du et al. [23] combined kernelized correlation filters (KCF) with three-frame differences to enhance small object detection and tracking performance. Wu et al. [24] considered slow motion and motion features, designing a nonmaximum suppression (NMS) module to assist in object detection. Ao et al. [25] used three-frame differences to produce binary difference images and employed a discriminative algorithm based on multimodal features and trajectory association to detect small moving vehicles. Feng et al. [26] proposed a cross-frame key point detection network (CKDNet) based on CornerNet, which enhances detection capabilities by extracting interframe information through a cross-frame module. Xiao et al. [1] developed a dual-stream network (DSFNet) for moving vehicle detection in satellite videos, where the static stream extracts single-frame contextual information, and the dynamic stream captures motion information across consecutive frames. Pi et al. [27] utilized motion information from adjacent frames combined with a transformer to estimate key points and predict scales, suppressing dynamic clutter and enhancing semantic feature extraction. While these multiframe motion-based methods integrate deep learning and satellite video characteristics, the direct input of multiframe data often results in interframe information overlaps, leading to the omission of weak targets in complex scenes and incurring high computational costs that reduce real-time performance.

Inspired by multiframe difference detection methods [1,26], this study proposes a moving vehicle detection method that integrates difference and convolution to fuse spatiotemporal information; additional details are introduced in Section 3.

## 3. Methods

**Figure 1:**
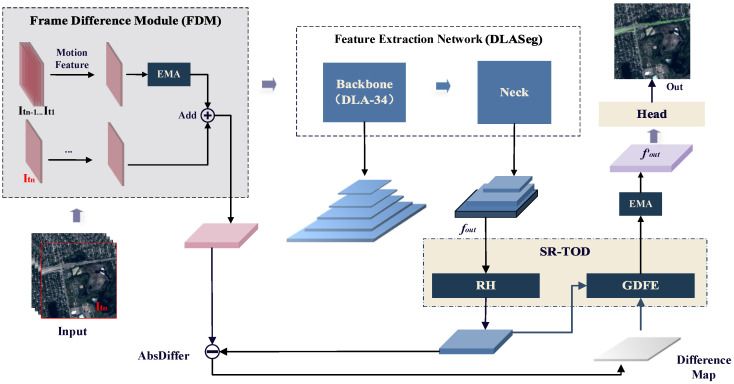
The general framework of the methodology is proposed in this paper.

In this paper, we propose a method for detecting moving vehicles in satellite videos by combining frame differencing and convolution, using the CenterNet [28] moving object detection network as the baseline architecture. The overall framework is illustrated in Figure 1.

The proposed method consists of three main components: the frame difference module (FDM), a feature extraction network, and the self-reconstructed tiny object detection (SR-TOD) framework. The head structure is consistent with CenterNet. The FDM takes the current frame and a sequence of consecutive historical frames as input. By leveraging frame differencing and convolution, it extracts the initial spatiotemporal features of vehicles in the current frame. The feature extraction network, DLASeg, is composed of the backbone network DLA-34 [29] and a neck module. Specifically, the backbone network is responsible for extracting rich feature information from the input initial spatiotemporal features, while the neck combines high-level semantic features with low-level detail features extracted by the backbone. This enhances detection accuracy and improves the ability to capture fine details of small targets. Finally, the SR-TOD framework includes two modules: the reconstruction head (RH) and the difference-map-guided feature enhancement (DGFE) module. These are designed to increase the overall sensitivity of the network to small targets and enhance the representation of their subtle features.

### 3.1. EMA Module

In practical applications, dynamic changes in target objects and backgrounds lead to variations in target positions, shapes, and other features across frames. Additionally, factors such as noise, illumination changes, and issues with acquisition equipment increase the complexity of target detection. When processing multiframe images, interframe interference often arises, resulting in information mixing.

Attention mechanisms offer a solution by automatically learning to focus on regions where target objects are located. These mechanisms dynamically adjust the weights of different features based on the contextual information in the current frame, enhancing features relevant to the target while suppressing those from the background or irrelevant regions. This approach reduces interference from extraneous information and mitigates the mixing of interframe data.

**Figure 2:**
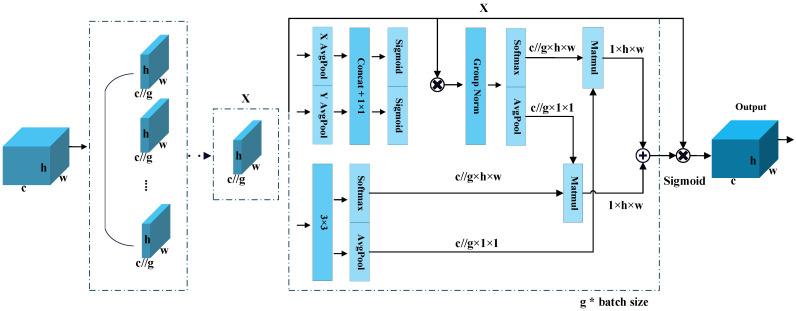
Efficient multiscale attention (EMA) module [30].

The efficient multiscale attention (EMA) module [30] eliminates the need to compute attention maps over the entire image. Instead, it reshapes parts of the channel dimension into the batch dimension and divides the channel into multiple subfeatures. By combining global information encoding and cross-spatial learning, the EMA module reduces computational overhead while preserving channel information. EMA has demonstrated strong performance in both image classification and object detection tasks. Compared to existing attention mechanisms, EMA offers advantages in terms of parameter efficiency and computational cost, while achieving higher detection accuracy. Therefore, EMA is embedded into the frame difference module and at the network’s output to reduce interframe information mixing and enhance the detailed features of vehicle targets. The detailed structure of the EMA module is shown in Figure 2.

### 3.2. Frame Difference Module

Due to the limitations of imaging spatial resolution, vehicle targets in satellite videos are tiny and lack distinct features such as texture and color. This makes it challenging for conventional deep learning methods to effectively detect moving vehicles with weak features. Since vehicle motion exhibits continuity, calculating the difference between adjacent image frames can capture changes between frames. However, traditional frame differencing methods rely on manually set thresholds to distinguish targets, which are difficult to adapt to varying scenes and are prone to false detections.

**Figure 3:**
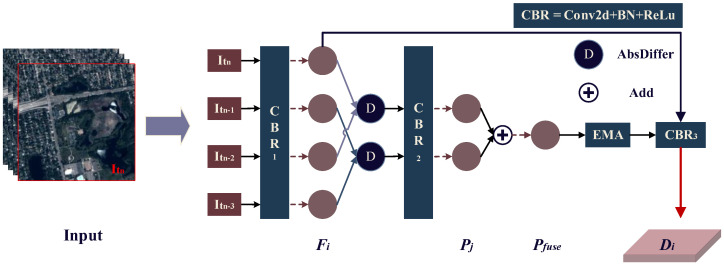
Frame difference module (FDM) architecture.

To address these challenges, this study introduces a frame difference module (FDM) that is integrated into the first layer of the feature extraction network. The FDM computes interframe differences on adjacent frames to extract temporal information and applies convolution to capture shallow spatial features. By fusing temporal and spatial information, it enhances the representation of target vehicles. The module is involved in backpropagation, allowing the convolution weights within the FDM to continuously learn and adapt as the model is trained. Compared to traditional frame difference methods, incorporating convolution into the frame difference method is more robust and effective for detecting moving vehicles in satellite videos. The structure of the FDM is shown in Figure 3.

For the continuous input frames $V=\left\{I_{t_{1}},I_{t_{2}},\ldots ,I_{t_{n}}\right\}$, $I_{t_{n}}$ indicates the current frame and the others indicate historical frames, and ∆*t* represents the interval between adjacent frames. Two dimensional convolution, batch normalization, and rectified linear unit (ReLU) (CBR) are performed on each frame individually to obtain the shallow spatial features, i.e., $F=\left\{F_{t_{1}},F_{t_{2}},\ldots ,F_{t_{n}}\right\}$. Using differences and convolution, the differential temporal features $P=\left\{P_{t_{1}},P_{t_{2}},\ldots ,P_{t_{n}}\right\}$ are derived from the shallow spatial features of consecutive frames. The detailed equation is illustrated below.

(1) ReLU(x)=max(0,x)

(2) $$F_{i}={\mathrm{CBR}}_{1}(I_{i}),\;i\in ((t_{1},t_{2},\ldots ,t_{n}))$$

(3) $$P_{j}={\mathrm{CBR}}_{2}(\mathrm{AbsDiffer}(F_{j},F_{j+2}),\;j\in ((t_{1},t_{2},\ldots ,t_{n-2}))$$

where *x* is the input to the hidden unit, AbsDiff(·) represents absolute difference, and *i* represents any one of the input multiple frames.

Summing each element in the differential temporal feature information $P=\left\{P_{t_{1}},P_{t_{2}},\ldots ,P_{t_{n-2}}\right\}$ and using the EMA module enhances the temporal information while suppressing background noise to obtain the fused temporal feature $P_{fuse}$. This is then fused with the shallow spatial feature $F_{t_{n}}$ to produce the fused spatial–temporal information feature $D=\left\{D_{t_{1}},D_{t_{2}},\ldots ,D_{t_{n}}\right\}$. This process highlights the feature information of the moving object across successive frames and enhances the current frame features, defined as

(4) $$P_{fuse}=\mathrm{EMA}(\mathrm{Add}(P_{j})),\;j\in ((t_{1},t_{2},\ldots ,t_{n-2}))$$

(5) $$D_{i}={\mathrm{CBR}}_{3}(\mathrm{Add}(F_{t_{n}},P_{fuse})),\;i\in ((t_{1},t_{2},\ldots ,t_{n}))$$

To ensure that the differential features reflect consistent motion and effectively capture moving vehicles, we designed FDM to use a fixed time interval and process a predefined number of input frames [18,23]. The historical frames are used solely to extract motion features and distinguish vehicles from the background.

### 3.3. Feature Extraction Network

The backbone of the proposed model leverages DLA-34 [29], a hierarchical deep neural network architecture with a tree-based feature aggregation structure. This design allows for flexible feature fusion paths, enhancing both semantic information and the detection of small objects. Compared to deeper and computationally intensive networks, DLA-34 achieves a favorable balance between accuracy and computational cost, making it well suited for detecting small objects in satellite videos.

**Figure 4:**
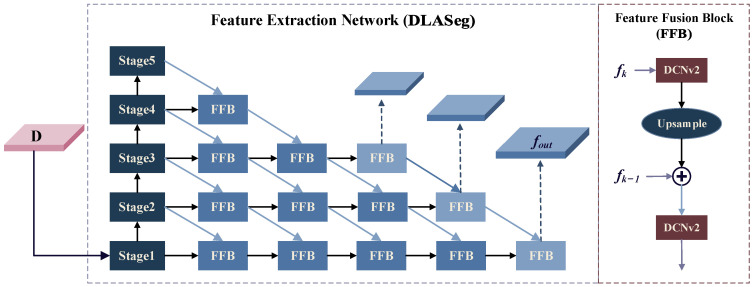
Feature extraction network architecture.

To effectively leverage both the detailed information from shallow features and the semantic richness of deep features, the neck employs an iterative deep aggregation and upsampling approach for progressive multiscale feature fusion. This process is implemented through a feature fusion block (FFB). The details of the FFB module are shown in Figure 4 and can be formulated as

(6) $$f_{k-1}=dc\left(dc\left(f_{k-1}\right)+up\left(dc\left(f_{k}\right)\right)\right)$$

where *dc*(·) denotes deformable convolution (DCNv2) [31] with a kernel size of 3 × 3, and *up*(·) denotes transposed convolutional layer. The deformable convolution used in FFB fuses the mismatched information between the low-level features and the high-level features. This fusion enhances the satellite features while maintaining accurate positioning and details, particularly for moving vehicles in the video.

### 3.4. Self-Reconstructed Tiny Object Detection Framework

**Figure 5:**
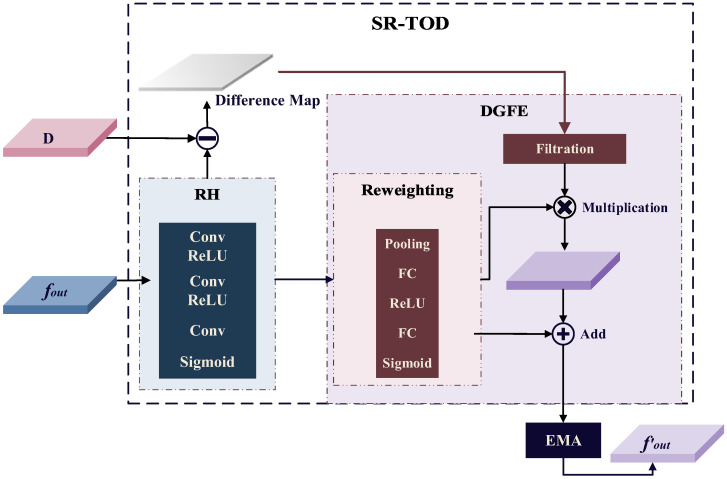
Self-reconstructed tiny object detection framework (SR-TOD).

The downsampling operations inherent in the backbone network inevitably result in information loss. This issue is particularly severe for small objects, as their limited size and sparse feature information amplify the loss. To address this challenge and enhance the network’s ability to represent small object features, the self-reconstructed tiny object detection (SR-TOD) framework [32] is introduced, the structural details of which are illustrated in Figure 5.

SR-TOD integrates an image reconstruction (RH) module between the neck and the detection head. It leverages shallow high-resolution feature maps, which contain more local details and positional information, to perform image reconstruction. The reconstructed images *f_rh_* can be computed as

(7) $$Conv_{i}=\mathrm{ReLu}(Conv(x)),i=1,2$$

(8) $$f_{rh}=Sigmoid(Conv(Conv_{2}(Conv_{1}(f_{out}))))$$

where *Sigmoid*(·) denotes the sigmoid function and *Conv* denotes the 2-D convolution.

Next, we compute a difference map between the reconstructed image and the initial input feature map *D* to activate the lost information of small target objects and provide potential a priori knowledge for detecting them. The formulation is

(9) $$\mathrm{DifferenceMap}=Mean_{channel}(Abs(f_{rh}-D))$$

where *Mean_channel_*(·) denotes computing the mean value along the channel dimension, and *Abs*(·) denotes computing the absolute value of each element.

In the difference-map-guided feature enhancement (DGFE) module, an element-wise attention matrix *M* is computed by reweighting the difference map along the channel dimension to obtain an enhanced feature map. This enhanced feature map is subsequently fed into the EMA module to further strengthen the features, producing *f*′*_out_*, which is then passed into the detection head for target detection. The specific formulas are

(10) Filtration(D)=Resize((Sign(DifferMap−t)+1)×0.5)+1

(11) $$M=Sigmoid(MLP(AvgPool(f_{rh}))+MLP(MaxPool(f_{rh})))$$

(12) $$f_{out}^{\prime }=\mathrm{EMA}(M⊗Filtration(D)+Filtration(D))$$

where *Sign*(·) denotes the sign function and *Resize* denotes resizing DifferMap to the same size as reconstructed images *f_rh_*. *AvgPool* denotes the average pooling along the spatial dimension, and *MaxPool* denotes the max pooling. The *MLP* includes two fully connected layers and a ReLU function.

## 4. Experimental Results and Analysis

### 4.1. Experimental Dataset

**Figure 6:**
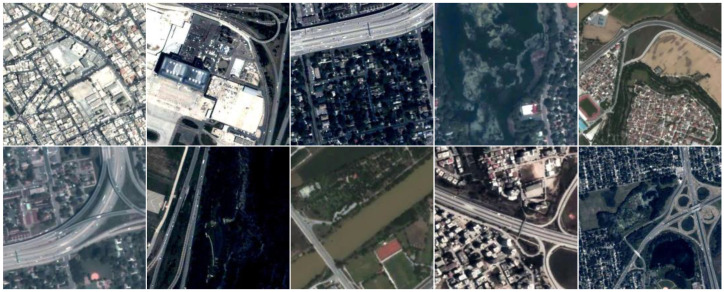
Sample presentation of the Jilin-1 satellite dataset.

The dataset used in this study was collected from the Jilin-1 satellite, which provides a ground sampling distance (GSD) of 0.92 m and a frame rate of 10 frames per second. The training set includes 72 videos comprising 27,434 image frames with a resolution of 512 × 512 pixels, while the test set consists of 7 videos containing 6716 image frames with a resolution of 1024 × 1024 pixels. A representative sample of the dataset is shown in Figure 6.

### 4.2. Experimental Setups

By default, the number of input frames to the network is set at *n* = 4, and the time interval between adjacent frames is Δ*t* = 1 and uses only the moving vehicles in the current frame as ground-truth labels. The batch size was set to 4, and the model was trained for 55 epochs using the Adam optimizer with an initial learning rate of 0.000124. The loss function is consistent with that of DSFNet [1]. All models were implemented on an NVIDIA RTX 4090 GPU.

### 4.3. Evaluation Metric

The following equations are used to quantitatively analyze the method provided in this article, considering precision, recall, and F1 score as quantitative evaluation measures:

(13) $$\mathrm{Precision}=\frac{\mathrm{TP}}{\mathrm{TP}+\mathrm{FP}}$$

(14) $$\mathrm{Recall}=\frac{\mathrm{TP}}{\mathrm{TP}+\mathrm{FN}}$$

(15) $$F1=2\times \frac{\mathrm{Precision}\times \mathrm{Recall}}{\mathrm{Precision}+\mathrm{Recall}}$$

where true positive (TP) represents correctly detected objects, false positive (FP) refers to false alarms, and false negative (FN) denotes missed detections.

Predictions are categorized as TP, FP, or FN based on the IoU (intersection over union) between the predicted bounding boxes and ground-truth bounding boxes. The tiny targets in satellite-video moving vehicle detection are highly sensitive to slight deviations in bounding boxes or center-point offsets, which can significantly impact IoU [33]. To address this, the conditions for determining TP were relaxed by setting the IoU threshold to 0.3 in this study. This threshold effectively balances moving vehicles’ localization accuracy and bounding-box fitting.

### 4.4. Performance Evaluations

To evaluate the superiority of the proposed algorithm in detecting vehicle targets in satellite videos, comparisons were conducted with seven mainstream methods, including traditional approaches (GoDec [10], DECOLOR [11], E-LSD [13], D&T [25], and B-MCMD [15]) and CNN-based methods (ClusterNet [21] and DSFNet [1]). The experimental results, presented in Table 1 and Table 2, show that the proposed method achieved the highest recall and F1 scores in six out of seven test videos and attained the highest average recall and F1 scores across all videos. Specifically, it surpassed the second-best DSFNet by 7.6% in recall and 3.9% in F1 score.

The performance of traditional methods is highly dependent on handcrafted features, which limits their ability to adapt to variations in real-world scenarios. As a result, these methods often exhibit low recall. The significant improvement over traditional methods can be attributed to the proposed algorithm’s ability to exploit spatiotemporal dynamic information, effectively capturing the temporal and spatial features of subtle moving objects. This capability suppresses false positives caused by the unstable satellite imaging platform and mitigates missed detections arising from dim lighting or complex backgrounds. As a result, the proposed method enhances detection performance and demonstrates robustness in challenging scenarios, such as those involving targets with low local contrast, weak texture features, or variable illumination conditions.

Compared to deep learning-based methods, the proposed approach not only achieves a notable improvement in recall but also enhances computational efficiency. The processing time per image is reduced by 7 s compared to the DSFNet model, achieving an effective balance between performance and efficiency. However, due to the SR-TOD framework’s emphasis on enhancing small object detection, some static targets may be misclassified, leading to a slight decrease in detection precision. Consequently, the proposed method ranks second to DSFNet in terms of average detection precision but achieves the best average F1 score. This means that SR-TOD still helps to improve small target detection performance.

**Figure 7:**
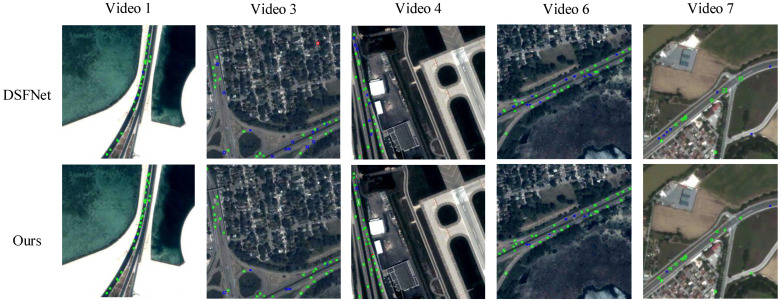
Results of different comparison methods in the test dataset. Green boxes: TP. Red boxes: FN. Blue boxes: FP.

A visual comparison of detection results between the proposed method and the DSFNet model is provided in Figure 7, where green, blue, and red boxes represent correct detections, missed detections, and false positives, respectively. As shown, moving vehicles occupy only a few pixels in complex backgrounds and are often surrounded by numerous distractors. The proposed method produces detection results comparable to those of the state-of-the-art DSFNet but with fewer missed detections and a stronger ability to identify hidden targets obscured by complex backgrounds. This demonstrates the proposed method’s superior robustness in challenging scenarios. For example, in Video 1, which features dynamic illumination changes, and Video 3, which contains numerous dim targets, the proposed method detects more missed targets than DSFNet. This improvement stems from its ability to model long-term spatiotemporal dynamics and learn subtle features embedded within complex backgrounds, facilitating the detection of dim and small moving targets in satellite videos.

### 4.5. Ablation Study

Ablation experiments were conducted using DSFNet and CenterNet as baseline models. The results, summarized in Table 3, highlight the impact of incorporating specific modules, with a checkmark (√) indicating the inclusion of the corresponding module.

**Figure 8:**
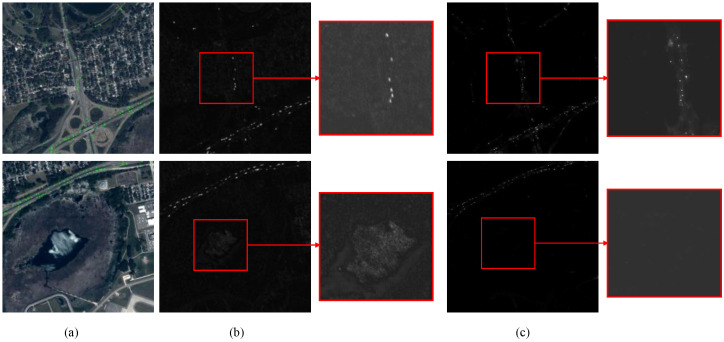
Visual comparison of the traditional frame difference method and the use of FDM. (**a**) Example of Jilin-1 satellite image (green dots represent ground truth). (**b**) Traditional frame difference method. (**c**) Using the frame difference module (FDM).

First, the frame differentiation module (FDM) was integrated into the CenterNet model. As shown in Table 3, adding FDM to CenterNet resulted in a 9.4% improvement in AP50 compared to the original model. This finding demonstrates that FDM effectively extracts motion features, thereby enhancing the model’s capability to detect moving targets. Moreover, unlike DSFNet, which employs 3D convolution for temporal information extraction at a high computational cost, FDM utilizes 2-D convolution, significantly reducing the computational complexity. A visualization comparing traditional frame differences and motion features extracted with FDM is presented in Figure 8. As depicted in Figure 8b, traditional frame difference introduces considerable background noise, making it difficult to accurately distinguish moving objects. In contrast, FDM captures temporal changes via differentiation and spatial semantic information via convolution, integrating the two to suppress noise and edge artifacts from buildings. This enables FDM to effectively isolate moving vehicles from the background, as illustrated in Figure 8c.

Subsequently, the efficient multiscale attention (EMA) module was incorporated into the FDM-enhanced CenterNet model. As reported in Table 3, the inclusion of EMA improved AP50 by 3.8% over the model without EMA. This improvement is attributed to EMA’s ability to alleviate frame-to-frame information interference without increasing the parameter count or computational load, thus further enhancing the network’s feature optimization capabilities.

Finally, the SR-TOD was integrated into the model with both FDM and EMA. While this addition led to a slight decrease in average precision (AP) compared to DSFNet and the model without SR-TOD, the resulting increase in average recall outweighed the drop in precision. This resulted in an improved average F1 score, which was 0.9% higher than that of the model without SR-TOD and 3.9% higher than DSFNet. SR-TOD significantly enhanced the model’s ability to detect objects with low local contrast and weak texture features while maintaining robustness against false positives introduced by the unstable satellite imaging platform. Additionally, the AP50 metric increased by 2% compared to the model without SR-TOD and by 0.9% compared to DSFNet, confirming that SR-TOD effectively contributes to improving the model’s detection performance.

## 5. Conclusions

In this paper, we propose a satellite video-based moving vehicle detection algorithm that leverages differential and convolution techniques to address the challenges posed by the small size and weak texture features of moving vehicle targets in satellite videos. Our approach employs FDM to extract spatiotemporal features from consecutive frames, enhancing motion feature representation and mitigating the limitations of single-frame images in capturing motion information. Within the detection network, deformable convolution and EMA modules are incorporated to preserve the integrity of information across consecutive frames. Additionally, SR-TOD is utilized to improve the representation of small targets. Experimental results show that our method achieves the highest recall among existing approaches while maintaining competitive detection accuracy in complex scenes. It also attains the highest F1 score, striking a balance between detection performance and speed, thereby meeting the demands of satellite video-based moving vehicle detection. Despite these advances, further research is needed to refine the processing of continuous frame information, particularly in integrating different differential features. Moreover, while SR-TOD excels in detecting static tiny targets, its performance for dynamic tiny targets requires further enhancement.

## Figures and Tables

**Table 1:** Recall (Re), precision (Pr), and F1 score achieved by different methods on the test dataset. The best results are shown in red, and the second-best results are shown in blue.

Method	Video 1			Video 2			Video 3			Video 4			Video 5			Video 6			Video 7		
	R (%)	P (%)	F1 (%)	R (%)	P (%)	F1	R (%)	P (%)	F1 (%)	R (%)	P (%)	F1 (%)	R (%)	P (%)	F1 (%)	R (%)	P (%)	F1 (%)	R (%)	P (%)	F1 (%)
**GoDec**	70.5	79.5	74.8	50.5	86.1	63.7	37.7	87.7	52.7	51.9	81.3	63.4	45.9	76.5	57.4	45.4	74.8	56.5	24.4	68.2	35.9
**DECOLOR**	29.4	99.7	45.5	52.8	90.4	66.6	45.9	86.9	60.1	32.4	98.9	50.9	51.5	88.8	65.2	55.5	77.5	64.7	21.1	89.1	34.1
**E-LSD**	58.8	77.4	66.8	40.5	41.4	40.9	36.2	92.4	52.0	46.5	90.2	61.3	35.6	87.3	50.6	34.0	81.8	48.1	37.8	74.8	50.2
**D&T**	59.3	93.1	72.4	43.0	89.8	58.2	40.1	87.3	54.9	59.4	83.2	69.3	36.7	85.8	51.4	41.1	81.2	54.6	60.9	56.9	58.8
**B-MCMD**	62.8	94.4	75.5	46.2	88.0	60.6	40.2	83.0	54.2	56.4	72.2	63.3	31.4	81.6	45.4	42.7	74.1	54.2	58.9	62.2	60.5
**ClusterNet**	61.9	65.1	63.4	41.7	86.8	56.3	44.9	78.8	57.2	41.1	74.2	52.9	43.7	85.7	57.9	51.4	80.8	62.8	53.4	82.2	64.7
**DSFNet**	76.9	94.9	85.0	58.5	96.3	72.8	50.5	94.6	65.9	70.3	98.2	81.9	73.9	95.4	83.3	53.9	96.2	69.1	46.3	98.0	62.9
**Ours**	80.9	90.6	85.4	67.6	94.6	78.8	61.0	90.8	73.0	80.6	92.5	86.2	70.4	94.1	80.5	64.0	93.8	76.1	59.0	80.9	68.2

**Table 2:** Average recall (AvgRe), average precision (AvgPr), average F1 score (AvgF1), and time cost (s) for a single frame (1024 × 1024) achieved by different methods on the test dataset. The best results are shown in red, and the second best results are shown in blue.

Method	AvgRe (%)	AvgPr (%)	AvgF1 (%)	Time Cost (s)
**GoDec**	46.6	79.2	57.8	5.10
**DECOLOR**	41.5	90.2	55.3	8.20
**E-LSD**	41.3	77.9	52.8	34.20
**D&T**	48.6	82.5	60.0	0.18
**B-MCMD**	48.4	79.4	59.1	45.70
**ClusterNet**	48.3	79.1	59.3	0.40
**DSFNet**	61.5	96.2	74.4	0.29
**Ours**	69.1	91.0	78.3	0.22

**Table 3:** Results on ablation experiments, the best results are shown in red, and the second-best results are shown in blue.

**Method**	**FDM**	**EMA**	**SR-TOD**	**Params (M)**	**FLOPs (G)**	**AP50**	**AvgRe (%)**	**AvgPr (%)**	**AvgF1 (%)**
**CenterNet**				6.62	41.61	54.3	-	-	-
**CenterNet**	√			6.62	45.70	65.8	-	-	-
**CenterNet**	√	√		6.62	45.70	69.6	66.5	93.3	77.4
**DSFNet**				6.71	53.90	70.5	61.5	96.2	74.4
**Ours**	√	√	√	6.63	45.70	71.6	69.1	91.0	78.3

## Data Availability

Publicly available datasets were analyzed in this study.
